# The prognostic value of tumor depth for cervical lymph node metastasis in hypopharyngeal and supraglottic carcinomas

**DOI:** 10.1002/hed.25667

**Published:** 2019-01-28

**Authors:** Lu‐Lu Ye, Jia Rao, Xing‐Wen Fan, Fang‐Fang Kong, Chao‐Su Hu, Hong‐Mei Ying

**Affiliations:** ^1^ Department of Radiation Oncology Fudan University Shanghai Cancer Center Shanghai China; ^2^ Department of Pathology Fudan University Shanghai Cancer Center Shanghai China; ^3^ Department of Oncology Shanghai Medical College, Fudan University Shanghai China

**Keywords:** cervical lymph node metastasis, hypopharynx, supraglottic larynx, surgery, tumor depth

## Abstract

**Background:**

To analyze the prognostic value of the clinicopathological parameters of primary lesions for predicting cervical lymph node metastasis in patients with hypopharyngeal and/or supraglottic carcinoma.

**Methods:**

We enrolled 127 patients with squamous cell carcinomas originating in the hypopharyngeal and/or supraglottic regions.

**Results:**

Multivariate analysis identified the tumor depth as an independent predictive factor for lymph node metastasis (odds ratio, 4.959; 95% confidence interval, 2.290‐10.739; *P* < 0.0001) with a predictive value of 0.966. A cutoff value of 4.5 mm was determined.

**Conclusion:**

The tumor depth of the primary lesion is a potent predictor of cervical lymph node metastasis in hypopharyngeal and supraglottic carcinomas. In cases with clinically negative nodal status, elective neck dissection should be adopted for patients with a tumor depth reaching 4.5 mm. Regular outpatient follow‐up is recommended for patients with a tumor depth less than 1.0 mm. Close follow‐up or preventative therapy should be considered between 1.0 and 4.5 mm.

**Abbreviations**
AUC: area under the curve
BM: basement membrane
CI: confidence interval
cN−: clinically negative nodal metastasis
cN+: clinically positive nodal metastasis
ECS: extracapsular spread
END: elective neck dissection
HPSCC: hypopharyngeal squamous cell carcinoma
N+: cervical nodal metastasis
pN: pathological nodal classification
pN‐: pathologically negative nodal metastasis
pN+: pathologically positive nodal metastasis
pT: pathological tumor
ROC: receiver operating characteristic
SGSCC: supraglottic squamous cell carcinoma

## 1 INTRODUCTION

Hypopharyngeal squamous cell carcinoma (HPSCC) and supraglottic squamous cell carcinoma (SGSCC) present with similar biological characteristics and frequent mutual invasion because these regions are in close proximity to each other. In addition to the hidden anatomical structures, extensive submucosal spread and early lymphatic invasion further give rise to advanced diseases at primary diagnosis, aggressive behavior, and poor outcomes.^1^, ^2^

HPSCC and SGSCC frequently present with initial cervical nodal metastatic disease (N+ disease)^1^, ^3^, ^4^, ^5^, ^6^, ^7^, ^8^, ^9^, ^10^ or with delayed regional metastasis.^1^ N+ disease is generally less responsive to treatment than the primary tumor.^11^ It has been claimed to be a more potent prognostic indicator than the tumor stage of the primary lesion and is associated with a higher risk of regional relapse and distant metastasis, and inferior survival in patients with HPSCC and SGSCC.^1^, ^2^, ^6^, ^12^

Surgical ablation and/or chemoradiotherapy are currently the accepted protocols for HPSCC and SGSCC.^13^ Primary tumor resection with cervical lymph node dissection remains the main approach for patients with clinically N+ (cN+) disease. The management of patients with clinically negative nodal metastasis (cN−) is controversial,^14^ because the phenomenon of occult metastasis is frequently detected.^8^, ^9^, ^10^, ^15^ The identification of objective prognostic determinants for cervical lymph node metastasis is of critical importance to enable better individualized therapy decisions. The objective of this study was to investigate the prognostic markers in surgical specimens to stratify a subset of patients with a high‐risk of N+ disease.

## 2 PATIENTS AND METHODS

### 2.1 Study population

This retrospective study was approved by the Institutional Review Board of Fudan University Shanghai Cancer Center. The study was performed in accordance with the principles of the Declaration of Helsinki and its amendments.

Between August 2007 and December 2016, the medical records of patients with primary HPSCC and SGSCC undergoing radical surgery at Fudan University Shanghai Cancer Center were reviewed. The eligibility criteria were as follows: (a) histologically confirmed squamous cell carcinoma in the hypopharyngeal or supraglottic region; (b) cervical nodal status initially evaluated with contrast‐enhanced MRI or CT preoperatively; (c) no preoperative chemotherapy or radiotherapy; and (d) no history of cervical lymph node dissection. Patients who were diagnosed with carcinoma in situ, had positive surgical margins, or recurrence were excluded.

Patients were screened with a full workup before treatment, including a complete medical history, physical examination, electronic laryngoscope examination, esophageal barium meal examination, contrast‐enhanced MRI or CT scan of the larynx, plain chest CT scan, abdominal ultrasound, whole‐body single‐photon emission CT bone scan, complete blood count, and serum biochemistry profile. The tumor stage was classified using the 7th edition of the American Joint Committee on Cancer staging system.

### 2.2 Surgery and adjuvant therapy

All patients underwent radical resection of the primary lesion and cervical lymph node dissection. Radical neck dissection was performed ipsilaterally in patients with cN+ disease, which involves levels II to VI or level I involvement. In case of contralateral cN− disease, elective neck dissection (END) for contralateral neck was carried out in patients with tumors approaching or crossing the midline of the sagittal plane or tumor arising from the posterior wall or postcricoid regions. With regard to bilateral cN−, ipsilateral END was adopted in patients with lesion in pyriform sinus unilaterally not approaching the midline. Otherwise, bilateral END was performed. The scope of END included levels II to IV or level VI involvement.

Postoperative radiotherapy was based on the pathological findings, including (a) a primary pathological tumor classification (pT classification) of 3 or above, (b) close margins (<5 mm), (c) a pathological nodal classification (pN classification) of 2 or above, (d) extracapsular spread (ECS) of the lymph node, (e) perineural invasion, and (f) lymphovascular invasion. Radiotherapy was administered in the form of intensity‐modulated radiotherapy with 6 MV photons. The prescribed dose was 1.8‐2.0 Gy in a daily fraction, given 5 days per week. The total dose was 66‐70 Gy to the gross target volume of primary lesion and metastatic lymph nodes, 60 Gy to the high‐risk microinvasive areas, and 54 Gy to the low‐risk areas. For patients with ECS, concurrent chemotherapy with cisplatin‐based agents was dosed at 80 mg/m^2^ every 3 weeks or 40 mg/m^2^ weekly.

### 2.3 Histopathological analysis

**Figure 1:**
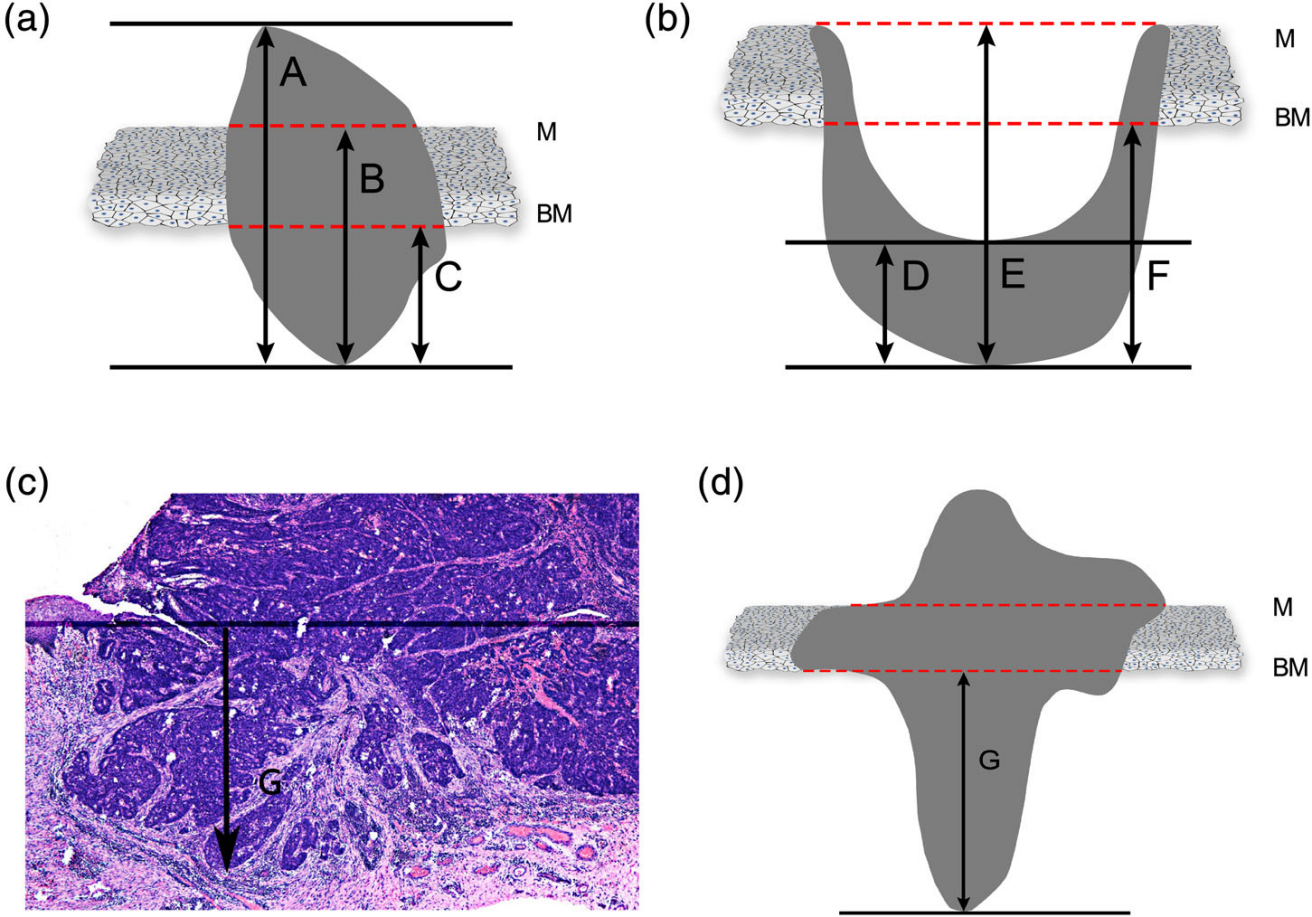
Measurement methods for histological evaluation: (i) Tumor depth (from basement membrane): dimension C in exophytic specimen (a) or dimension F in ulcerative specimen (b); (ii) tumor depth (from mucosal surface): dimension B in exophytic specimen or dimension E in ulcerative specimen; (iii) tumor thickness: dimension A in exophytic specimen or dimension D in ulcerative specimen. For specific histological evaluation (c; d was the animated version of c), dimension G was taken as tumor depth. Abbreviations: BM, basement membrane; M, mucosal surface [Color figure can be viewed at wileyonlinelibrary.com]

All surgical specimens were oriented and labeled by the surgeons before fixation in 10% buffered formalin and embedded in paraffin. The specimens were sectioned for routine hematoxylin and eosin staining. The histopathological review was performed by two experienced pathologists who were blinded to the patients' medical information. When significant disagreement occurred, a third pathologist was needed to minimize the deviation. There was wide variation in the manner how tumor depth was measured^16^ in the literature which was illustrated in Figure 1. In our study, the tumor depth of invasion was measured from the deep surface of the basement membrane (BM) to the deepest aspect of the tumor. If the tumor was exophytic, the tumor depth is equivalent to dimension C (Figure 1a). While in ulcerative tumors, dimension F was taken (Figure 1b). In a specific case (Figure 1c,d) that was eligible for our research, the bold arrow (dimension G) indicates tumor depth.

### 2.4 Statistical analysis

The Statistical Package for the Social Sciences version 22.0 (IBM, Armonk, New York) was used for the data analysis. The chi‐square test or Fisher's exact test was performed to determine the correlation between the categorical variables and lymph node metastasis. The Mann‐Whitney *U* test was carried out to assess the relationship between numeric variables and lymph node metastasis. A multivariate logistic regression model with a stepwise selection method was used in the analysis. Analysis of variance was performed to compare the means of more than two populations. Any result with a two‐sided *P*‐value <0.05 was considered to be statistically significant. The area under the curve (AUC) calculated from the receiver operating characteristic (ROC) analysis was applied to evaluate the predictive abilities of the variables and the optimal cutoff value.

## 3 RESULTS

### 3.1 Basic characteristics

A total of 127 patients were enrolled in the final analysis. The majority of the patients were men (97.6%). The mean age was 57.1 ± 9.2 years old. Of these patients, 93 (73.2%) had HPSCC and 34 (26.8%) had SGSCC. The affected subsites of the hypopharynx were the pyriform sinuses in 81 cases, the postcricoid area in 6 cases, and the posterior pharyngeal wall in 6 cases.

Table 1 shows the distribution of cases according to the clinical and pathological evaluation of cervical nodal status. There were 32 (25.2%) and 95 (74.8%) patients who had cN− and cN+ status, respectively, while 102 (80.3%) patients had pathologically confirmed positive nodal metastasis (pN+) with 83.9% (78/93) for HPSCC and 70.6% (24/34) for SGSCC cases. The overall incidence of occult nodal metastasis was 37.5% (12/32) and was 35.3% (6/17) for HPSCC and 40% (6/15) for SGSCC. In terms of pT classification, the risk of N+ disease was 70% (14/20) in pT1, 80.6% (54/67) in pT2, 88.9% (16/18) in pT3, and 81.8% (18/22) in pT4 disease. There was no significant difference in the distribution of pN+ disease (*P* = 0.53).

**Table 1:** The distribution of cases according to clinical and pathological cervical evaluation of nodal status

	pN status						Total number
cN status	N0	N1	N2a	N2b	N2c	N3	
N0	20	9	0	3	0	0	32
N1	5	18	1	24	3	0	51
N2a	0	0	0	3	0	0	3
N2b	0	1	0	21	8	1	31
N2c	0	2	0	3	4	0	9
N3	0	0	0	0	0	1	1
Total number	25	30	1	54	15	2	127

Abbreviations: cN, clinical nodal metastasis; pN, pathological nodal metastasis.

### 3.2 Association between clinicopathological factors and cervical nodal status

In the univariate analysis, tumor depth (*P* < 0.0001) and lymphovascular invasion (*P* = 0.02) showed significant correlations with N+ disease (Table 2). After the multivariate logistic regression analysis adjusted for tumor sites and biological variables, tumor depth remained a significant risk factor for nodal metastasis (odds ratio, 4.959; 95% confidence interval [CI], 2.290‐10.739; *P* < 0.0001; Table 3). The overall predictive value of the tumor depth for nodal metastasis was 0.966 and was 0.971 for HPSCC and 0.983 for SGSCC, respectively.

**Table 2:** Univariate analysis of clinicopathological risk factors for cervical nodal metastasis

Variables	pN status		
	pN− (No. = 25)	pN+ (No. = 102)	*P* value
Age (mean ± SD, years)	59.4 ± 9.3	56.6 ± 9.1	0.18^a^
Sex, n (%)			
Male	25 (100.0)	99 (97.0)	
Female	0 (0.0)	3 (3.0)	
Tumor site, n (%)			0.1^b^
Hypopharynx	15 (37.0)	78 (76.0)	
Supraglottis	10 (63.0)	24 (24.0)	
pT classification^c^, n (%)			0.53^b^
T1	6 (24.0)	14 (13.7)	
T2	13 (52.0)	54 (53.0)	
T3	2 (8.0)	16 (15.7)	
T4	4 (16.0)	18 (17.6)	
Histological grading, n (%)			0.26^b^
G1	2 (7.4)	3 (3.0)	
G2	19 (77.8)	70 (68.0)	
G3	4 (14.8)	29 (29.0)	
Perineural invasion, n (%)			1.000^b^
−	22 (88.0)	88 (86.3)	
+	3 (12.0)	14 (13.7)	
Lymphovascular invasion, n (%)			**0.02** b
−	24 (92.6)	77 (76.0)	
+	1 (7.4)	25 (24.0)	
Maximal tumor diameter (mean ± SD, mm)	28.4 ± 13.0	31.0 ± 14.2	0.4^a^
Tumor depth (mean ± SD, mm)	2.0 ± 1.3	7.4 ± 3.0	<**0.0001** a

Abbreviations: CI, confidence interval; G1, well differentiated; G2, moderately differentiated; G3, poorly differentiated; OR, odds ratio; pN−, pathologically negative nodal metastasis; pN+, pathologically positive nodal metastasis; pT Classification, pathological tumor classification.

Bold values show *P*‐value<0.05.

a Mann‐Whitney *U* test, *P* < 0.05.

b Chi‐square test or Fisher's exact test, *P* < 0.05.

c Tumor node metastasis staging system according to the American Joint Committee on Cancer (7th edition).

**Table 3:** Multivariate logistic regression analysis of variables for cervical nodal metastasis adjusting for tumor sites

Variables	OR (95% CI)	*P* value	AUC (95% CI)
Maximal tumor diameter	0.979 (0.902‐1.063)	0.61	0.543 (0.416‐0.671)
Tumor depth	4.959 (2.290‐10.739)	<**0.0001**	0.966 (0.939‐0.993)
Lymphovascular invasion	12.911 (0.733‐227.551)	0.08	0.603 (0.490‐0.715)
Histological grading	2.681 (0.369‐19.462)	0.33	0.578 (0.456‐0.701)
Perineural invasion	0.405 (0.013‐12.778)	0.61	0.509 (0.383‐0.634)

Abbreviations: AUC, area under curve; CI, confidence interval; OR, odds ratio.

Bold value shows *P*‐value<0.05.

The mean tumor depths in pT1 (4.2 ± 2.4 mm), pT2 (6.0 ± 2.9 mm), pT3 (7.5 ± 3.2 mm), and pT4 (8.3 ± 4.7 mm) were different (*F* = 6.431, *P* < 0.0001). The pT1 group was found significantly different from the other pT groups, whereas no differences in tumor depths among the pT2, pT3, and pT4 groups.

In terms of cervical nodal status, the mean tumor depths were 2.0 ± 1.3 mm (range, 0.6‐4.4 mm) in the pN− group and 7.4 ± 3.0 mm (range, 1.2‐15.0 mm) in the pN+ group. This difference was statistically significant (*t* = 13.837; *P* < 0.0001). Similarly, the mean tumor depths in the pN− (2.0 ± 1.3 mm), pN1 (6.4 ± 2.9 mm), pN2 (7.8 ± 3.0 mm), and pN3 (7.1 ± 3.5 mm) groups were different (*F* = 28.370, *P* < 0.0001). The pN− group was different from each pN+ stage group, yet no differences in tumor depths were found among the pN+ groups.

**Figure 2:**
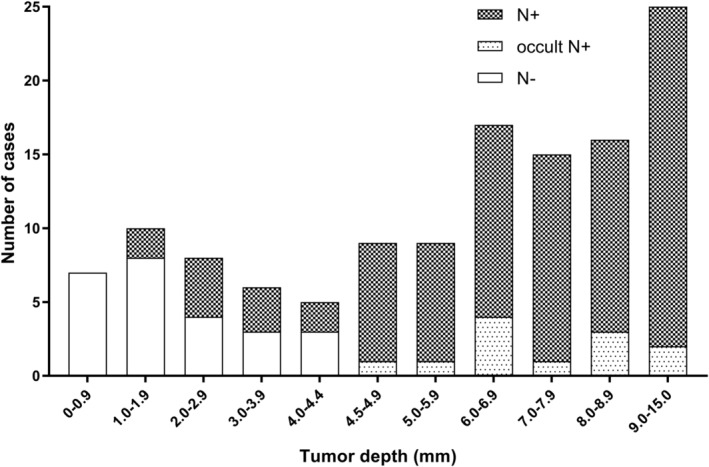
The distribution of pathological nodal status in terms of the range of tumor depth. (a) N+: clinically and pathologically positive nodal status; (b) occult N+: clinically negative nodal status with pathologically positive confirmation; (3) N−: clinically and pathologically negative nodal status

Figure 2 shows the pN status corresponding to the range of tumor depth. No nodal metastasis (0/7) was found when the tumor depth was less than 1.0 mm, and 20% (2/10) of metastasis was seen with the tumor depth between 1.0 and 2.0 mm. It reached 50% when the tumor depth exceeded 2.0 mm. The rate showed a rising trend with increasing tumor depth and was 100% with the tumor depth in excess of 4.5 mm. With regard to the occult nodal disease, none was found until the tumor depth reached 4.5 mm.

### 3.3 The cutoff value of tumor depth

ROC curve analysis was performed to evaluate the optimal cutoff value of tumor depth for cervical nodal metastasis. The AUC were 0.892 (95% CI, 0.939‐0.993; *P* < 0.0001). A cutoff value of 4.5 mm (sensitivity: 89.2%, specificity: 100.0%) was chosen.

## 4 DISCUSSION

The incidence of initial N+ disease was previously reported to be 56.7%‐83.7% in HPSCC and 39.5%‐70% in SGSCC.^1^, ^3^, ^4^, ^5^, ^6^, ^7^ However, surgical data from END have demonstrated a high risk of occult nodal metastasis of 31%‐57% in HPSCC and 10%‐18% in SGSCC.^8^, ^9^, ^10^ Moreover, the incidences of delayed regional metastasis in HPSCC (18.5%‐31.1%) and SGSCC (16%) were directly related to the initial N+ classification and were tripled in advanced nodal disease. As a consequence, the probability of distant metastasis rises proportionally with the occurrence of initial or delayed nodal disease.^1^ Nodal disease demonstrates a major problem in deteriorating survival^1^, ^2^, ^6^, ^11^, ^12^ and highlights the pivotal need for accurate diagnosis and therapeutic control.

The anatomical characteristics and biological behaviors of the primary tumor are the chief determinants of the cervical nodal status. The tumor size is defined as the two‐dimensional surface area of the primary tumor and is currently used in the T classification. The tumor depth is considered as a reflection of the third dimension of the tumor reflecting the deepest structure that the tumor reached. It is well established that larger tumors are associated with worse survival than smaller tumors. On the other hand, it is believed that the prognosis tends to be inferior in vertically growing tumors compared with that in horizontally growing ones with same surface area.^17^ In short, T classification should be extended to include the third dimension of the tumor, that is, its vertical dimension.

Notably, tumor depth is not merely a quantitative assessment of the primary tumor but also a qualitative reflection of its aggressive potential for local infiltration. There are strong barriers in the human body against deep invasion of tumors. Tumor cells with a greater malignant potential are prone to break through these protective barriers and invade vertically. Horizontal spread, on the other hand, occurs in superficial lesions that are under the control of body resistance.^17^, ^18^

Although several studies^7^, ^17^, ^19^, ^20^ have considered tumor thickness and tumor depth synonymous, they are in reality different and should be distinguished. The tumor thickness refers to the thickness of the entire tumor mass, whereas the tumor depth is the extent of tumor growth into the tissue beneath the epithelial surface. With deeper invasion, tumors extend proximal to blood vessels, lymphatics and nerves, and the possibility of nodal metastasis rises accordingly.^16^ This distinction is particularly significant in exophytic tumors in which taking tumor thickness as an evaluating parameter may overestimate the actual invasion depth (dimension A; Figure 1a), whereas in ulcerative cases, it is an underestimating evaluation (dimension D; Figure 1b). The difference between the depth from the mucosal surface and the depth from the BM reflects the thickness of the normal mucosa, which usually remains constant (dimension B vs C, E vs F; Figure 1). In our study, we defined the tumor depth as the distance from the BM to the deepest point of invasion, as it seems to be a matter of course that lymph node metastasis occurs on the premise of the tumor spreading across the BM.

From the viewpoint of reliable detection of N+ disease, tumor depth has been previously identified in many malignancies. In terms of HNSCC, the significant importance of tumor depth for predicting N+ disease in oral cavity squamous cell carcinoma has been specified in the National Comprehensive Cancer Network Clinical Practice Guidelines.^21^ However, the significance in HPSCC and SGSCC was scarce.

Ambrosch et al.^22^ first reported that the tumor depth was the only risk factor for nodal disease in upper aerodigestive tract cancers. However, the obtained cutoff value of 4 mm was based on oral cancers in the majority of cases. Taner et al.^4^ indicated that the incidence of N+ disease in laryngeal cancers was significantly higher with a tumor depth exceeding 3.25 mm. Masayaki et al.^5^ analyzed 40 pharyngeal cases and recommended that END be considered for patients with a tumor depth greater than 1 mm. Nevertheless, the cervical nodal status was not sufficiently determined in each patient. Taniguchi et al.^19^ reached a similar conclusion. However, more than half of the cases were pathologically confirmed as carcinoma in situ, and it is commonly acknowledged that lymph node metastasis would not occur in such conditions. Even of 75 pharyngeal cancers with subepithelial invasion, the constitution ratio of the tumor sites was not mentioned.

The results of our study were in accordance with previous findings except for the cutoff value of tumor depth. This phenomenon can be attributed to the inclusion of different T classification. The majority of enrolled cases in Taner's study^4^ were early T classification cases. Masayaki and Taniguchi^5^, ^19^ collected specimens by endoscopic mucosal resection, implying that the included patients were restricted to those with relatively small and superficial lesions. We surmise that this was an inappropriate approach to exclude cases with advanced T classification from the analysis, because the correlation between T classification and nodal status was undefined.^7^, ^19^ There was no major difference in the distribution of N+ disease as to pT classification in our study.

As in our study, patients with a tumor depth less than 1.0 mm were found no nodal metastases. In addition to established postoperative therapy, regular outpatient follow‐up is recommended afterward in this low‐risk subgroup. Patients with a tumor depth equal to or greater than 4.5 mm were at high risk for N+ disease with the metastatic risk of 100%. The presence of occult nodal disease was detected in the condition of the tumor depth ≥ 4.5 mm. Therefore, in the cases of cN− status, END should be adopted in patients with a tumor depth reaching 4.5 mm. Eleven of 29 cases (37.9%) with a tumor depth between 1.0 and 4.5 mm (1.0 mm ≤ tumor depth < 4.5 mm) were found to have nodal metastasis. For this subgroup with moderate potential for N+ disease, the appropriateness of END must be determined by clinical judgment, because routine END is beneficial in less than 25% of the population.^23^ In addition, Dadas^20^ emphasized the reliability of frozen sections for determining tumor thickness intraoperatively. This is of much concern to facilitate surgeons' decision making for END.

The present study had several strengths distinguishing it from previous investigations. Each patient was uniformly assessed and treated with a standardized protocol that was unified by a multidisciplinary team discussion. Eligible cases were selected with strict grouping criteria. It is noteworthy that we carried out this study with the largest population sample size to date.^4^, ^9^, ^15^, ^22^ Concomitantly, we performed a multivariate analysis adjusting for tumor sites and further subgroup analysis to evaluate the correlation between tumor depth and pathological classification. Despite it was inadequately powered to demonstrate nodal classification, our study raised sufficient questions for further research to be validated in large populations, because we previously confirmed the predictive value of nodal burden for survival in HPSCC.^6^ It is recognized that the major limitation of this study was its retrospective nature from a single institution and associated biases. In this regard, a large prospective clinical trial in multicenter setting should be conducted for further evaluation.

## 5 CONCLUSION

It is of vital to understand the intrinsic mechanism of lymph node metastasis in HPSCC and SGSCC to provide crucial insights into cancer growth and consequently allow more rational therapeutic decision making in clinical practice. Tumor depth provides additional information in an effort to predict nodal status, in addition to that provided by traditional T classification and biological variables.

## CONFLICT OF INTEREST

The authors have no conflicts of interest to report.

## ETHICAL APPROVAL

All procedures performed in studies involving human participants were in accordance with the ethical standards of the institutional research committee and with 1975 Helsinki declaration and its later amendments or comparable ethical standards.

## AUTHOR CONTRIBUTIONS

All authors critically read the drafts of this paper and approved its final version prior to submission for publication.

*Study design*: Ye and Rao

*Data collection*: Fan and Kong

*Analysis and interpretation of the data*: Ye, Fan, and Kong

*Manuscript drafting*: Ye and Rao

*Manuscript revision*: Ye, Rao, Fan, Kong, Hu, and Ying.
